# Women psychiatrists in India: A reflection of their contributions

**DOI:** 10.4103/0019-5545.69277

**Published:** 2010-01

**Authors:** Mamta Sood, Rakesh K. Chadda

**Affiliations:** Department of Psychiatry, All India Institute of Medical Sciences, New Delhi - 110 029, India

**Keywords:** India, psychiatrists, women

## Abstract

The increasing number of women joining psychiatry is a relatively new phenomenon in the field of medicine. Keeping with the trends world over, the number of women psychiatrists in India has been on the rise over the last two to three decades. The authors searched various volumes of the Indian Journal of Psychiatry, recent membership directories of the Indian Psychiatric Society, website of the Medical Council of India and personal communications for contributions of the women psychiatrists in India. Women psychiatrists have a number of contributions to their credit in India. They have played important roles in the affairs of national professional organizations like the Indian Psychiatric Society and have contributed to the psychiatry education and research. However, they also suffer limitations because of the absence of adequate institutional support and policies looking into their specific needs.

## INTRODUCTION

There has been a significant increase in the number of women doctors joining psychiatry in India in the last two to three decades. Until early 1980s, their number could be counted in single digit. Historically, the discipline of psychiatry has often been considered to be associated with unpredictable and violent patients, and had a number of misconceptions attached to it; trends are changing now. Reasons for psychiatry becoming a preferred career choice for women doctors can be traced to multiple factors. Psychiatry has gradually shifted out of the high walls of the mental hospitals to the general hospital and community settings and is gaining more acceptability and respect in the society. The medical profession is also synonymous with long years of training followed by long hours at work almost every day. Over and above this, women doctors have to juggle career and family responsibilities as domestic and child related duties have largely remained with them despite changing roles. The crucial career-intensive years in professional life (residency and early years as junior faculty) coincide with the equally crucial period (marriage, childbearing and child rearing) in the personal life. A career in psychiatry allows doing justice to multiple roles in a better way than the other busy clinical specialties like gynecology and obstetrics, pediatrics, internal medicine, cardiology or surgical disciplines, because of the possibility of predictable working hours, less emergencies, flexible working schedules and greater opportunities to interact with patients.[1]

The increasing trends of psychiatry as a specialty choice for women doctors in India have remained in tandem with trends world over, albeit late by about a decade. For example, in the USA, psychiatry has the fourth highest number of women specialists and 40-45% of first-year residents in psychiatry are women.[2] In the UK and Ireland, women form 45-48% of the specialist trainees.[3] In Canada, the percentage of women psychiatric residents increased from 23.5% to 43.4% over a period of 10 years from 1970s to 1980s.[4]

In contrast to the high income countries where various issues related to women psychiatrists like their numbers, needs and concerns, defining characteristics and reasons for their lagging behind men have been researched and debated, there is virtual lack of data about the issues related to women psychiatrists in India.[5] Research has documented that their working styles have been noted to be different from their male colleagues in some aspects. Many women psychiatrists are noted to be more empathic in approach. Their patients report better satisfaction levels as they are more likely to engage patients as active partners in the care by adopting a democratic style of communication. They spend a significantly greater proportion of time on preventive services and counseling, compared to their male colleagues.[6] As a group, they lag behind their men colleagues in attaining positions of authority and leadership in academics, professional organizations, and medical institutions. For example, women constitute 29% of associate professors, 15% of full professors, and 6% of department chairs in medical schools in USA.[7] In England also, women psychiatrists are significantly less likely to pursue an academic career and professional position than men.[8] There are some reports that the women psychiatrists have poorer coping skills, more physical and emotional symptoms, and are more likely to report stress, anxiety and depression.[9]

The present paper aims at bringing out contributions made by Indian women psychiatrists to the field of psychiatry and society. The paper presents information available on women psychiatrists in India in terms of their numbers, concerns and research interests, important positions held by them in academia and various national bodies.

## MATERIALS AND METHODS

Indian Psychiatric Society, the national body of psychiatrists in India, is more than 60 years old. It publishes a directory of its members periodically. Membership directories of the Society, published in 2006 and 2009,[10,11] and all the volumes of Indian Journal of Psychiatry (1958-2009), and Indian Journal of Neurology and Psychiatry (1949-1953), the official publication of Indian Psychiatric Society were searched manually for the articles published, with women as first author. The official website of Medical Council of India was also searched for relevant information.[12]

## RESULTS AND DISCUSSION

### Number of women psychiatrists in India

Women psychiatrists constitute 14.6% of the total membership (2829) of Indian Psychiatric Society.[10] They constitute about 10% of the fellows and 20% of the ordinary members; a member of the society becomes eligible for fellow after five years post qualification experience in the psychiatry. These figures suggest that the number of women at relatively senior level is less. It is possibly due to the fact that the number of women doctors joining psychiatry has started increasing only recently as suggested by higher percentage of members with less than five years post qualification experience.

Women psychiatrists are represented in different sectors like general hospital psychiatric units, psychiatric hospitals and the office based practice. Exact figures of the gender based distribution of psychiatrists, in different settings, is not available. Although every year, 134 posts are available for postgraduate degree and 96 for postgraduate diploma in psychiatry,[12] it is not known how many women join these postgraduate courses every year. However, women have formed about 20% of the candidates joining psychiatry residency in the last five years at the All India Institute of Medical Sciences, New Delhi (authors’ medical school), one of the premier medical institutions of the country. These figures are not different from the total percentage of women doctors joining the institute for post graduation in various medical and surgical disciplines in the last five years.[5] It may not be appropriate to generalize these figures, but these provide some trends.

Most of the premier medical schools of the country have women faculty; a few of them are professors. However, most of them are at relatively junior positions. It is not known, how many have been heads of departments of psychiatry in different medical colleges or hospitals across the country. We could identify only a few. A few women have also been known to have headed a psychiatric hospital or a medical college.

### Indian Psychiatric Society

Currently, there is no female office bearer in the Indian Psychiatric Society and only one of the council members is a woman. However, since its inception in 1947, women psychiatrists have held the post of president four times: Prof. Ajita Chakraborty in 1976, Prof. Roshan Master in 1981, Prof. Jaya Nagaraja in 1983 and Prof. Deepali Dutta in 1990. Prof. Ajita Chakraborty has also held the post of general secretary of the society in 1967 and 1968. Though the society has been publishing its journal, the Indian Journal of Psychiatry, for more than 50 years, it never had a woman editor. In the current editorial board, there is no woman psychiatrist; only one is in the journal committee.[10] At present, one woman is the chairperson of the Society’s committee on psychiatry education. Three women are either chairpersons or conveners of the Society’s task forces on women and mental health and suicide prevention.[11] Many women psychiatrists have received national as well as international recognitions.

### Indian Journal of Psychiatry[5,13–183]

We were able to retrieve about 170 papers, which had women as first authors. There were only 45 papers published prior to 1980. However, we could identify only a limited number of women psychiatrists, who had published before 1980. They have contributed original papers, critiques, reviews and book reviews. The area of research is varied and includes psychopathology, child psychiatry, culture specific syndromes, neuroses, psychopharmacology and psychological testing. The women from allied branches like psychology, social work, occupational therapy and psychiatry nursing have also published in the journal. Interestingly, the women psychiatrists, who have contributed to the journal, were mostly from big cities, and almost all belonged to medical schools.

Probably, Dr. (Miss) Mani B Ghamat was the first woman psychiatrist to publish an article in the earlier version of the journal, Indian Journal of Neurology and Psychiatry in 1952.[65] She worked as assistant psychiatrist at JJ Hospitals, Bombay. She was also probably the first women psychiatrist recorded to be a member of the society. Prof. Ajita Chakarborty, Prof. Roshan Master, Prof. Erna Hoch, Prof. M. Sarada Menon, Prof. Deepali Dutta were some of the pioneer women researchers. Prof. Ajita Chakarborty has published extensively on epidemiology, culture specific issues in mental health, child psychiatry and psychopathology. Prof. Roshan Master wrote about psychopathology and psychopharmacology. She also wrote many excellent book reviews. Prof. Erna Hoch wrote about many important social and cultural issues. Prof. M. Sarada Menon contributed to research on psychopharmacology and was also instrumental in setting up the Schizophrenia Research Foundation (SCARF) at Chennai, which has been a trendsetter non-governmental organization (NGO) in the field of mental health. She worked extensively on rehabilitation of schizophrenia. Prof. Deepali Dutta wrote about the issues related to child mental health. Interestingly, Smt Shakuntala Pranjapye, one of the authors in the journal in 1963, was a member of legislative council of Maharashtra.[125]

The number of women psychiatrists who have published in the journal started increasing from 1980 onwards. Women from all types of work settings like medical colleges, private practice and services have contributed to the journal. It was a pleasant surprise that now woman psychiatrists not only from metros but even from smaller cities have also been publishing. Majority of the papers are original articles and some are case reports. However, reviews, invited articles, presidential addresses, editorials, commentaries, orations and critiques by women authors in the journal could be counted on fingers and were less than five in each category.

Interestingly, in the journal, only one woman psychiatrist has published more than 10 times, 17 have published twice, 10 have published less than 10 articles each and rest have published only once. This highlights the fact that women psychiatrists do publish initially but for reasons not researched yet, start leaving the academics. The reason for not publishing is apparently not due to lack of interest. It is possible that similar trends may be found for male psychiatrists. On pursuing the topics researched, it becomes apparent that they have interest in wide ranging subjects of psychiatry from biological to psychosocial underpinnings of various psychiatric disorders, epidemiology, biopsychosocial treatments, consultation liaison psychiatry, child psychiatry, suicide, severe and common mental disorders and de-addiction. A few women psychiatrists of Indian origin, now settled abroad, have also contributed to the journal.

The areas where women psychiatrists have established themselves as leaders are child psychiatry, schizophrenia, disability, suicide and mental health issues related to women.

### Other issues

There are no studies available comparing women and men psychiatrists in terms of their specific needs, aspirations, areas of interest, monetary incentives, working styles, characteristics and other issues. There are no guidelines for pregnancy and maternity leave for women doctors and post graduate trainees from the Medical Council of India, the regulatory body for medical matters in India. A post graduate trainee has to attend not less than 80% of the training during each calendar year. If pregnancy occurs during the period of postgraduate training, either the resident has to manage within the permitted time of not attending or has to postpone her examination. For those in a government sector job, there is a provision for maternity leave of 180 days.[184] Only a few hospitals or medical colleges provide reliable onsite daycare. School-based childcare is usually not available when children are older. On discontinuation of a job for family-building or other reasons, options for revival of career after a certain period are presently unavailable due to restrictions in age of entry and qualification for jobs at different levels. Currently there are no regulations or policies from government which address these issues. There is little opportunity for flexible training, creative scheduling of job or training if a woman wants to come back to academics after a gap due to child care responsibility. Unlike some of the high income countries, there is no central or state organization/union/association of women psychiatrists at regional or national level in India.

Although the country is credited for having the first woman Prime Minister in the region, currently there are very few women psychiatrists in top positions of the Indian Psychiatric Society or other national bodies despite their increasing numbers. Therefore, at present, the role of women psychiatrists in policy making of the specialty remains negligible. This is in contrast to earlier times when although women psychiatrists were less in number, they held important positions in the Indian Psychiatric Society. The gender inequity in promotion has been explained by the fact that women, because of family obligations, work fewer hours, are less productive, and have a limited publication record. Women have often fewer mentors and professional networks and less collegial support while in the academic medical system.[185]

We have attempted to access information about Indian women psychiatrists from multiple sources, and have presented the information whatever we could get. We offer our apologies if we have missed some important contributions of the Indian women psychiatrists.

## CONCLUSION

The number of women psychiatrists in India is on the rise in the recent years. Indian women psychiatrists have many contributions to their credit. Many of them have served or are serving as faculty in medical schools and have also contributed significantly to psychiatry education at undergraduate and postgraduate level. They have left their marks on various areas of psychiatry; significant among them are child psychiatry, suicide and its prevention, community psychiatry, rehabilitation of patients with schizophrenia and issues related to women mental health. But to succeed and flourish, they need support systems both at home and workplace. The profession and the government have to innovate in finding ways to maximize the optimal use of the substantial talent pool and intellectual capital of women psychiatrists.
